# Pharmacokinetic Drug–Drug Interactions and Herb–Drug Interactions

**DOI:** 10.3390/pharmaceutics13050610

**Published:** 2021-04-23

**Authors:** Min-Koo Choi, Im-Sook Song

**Affiliations:** 1 College of Pharmacy, Dankook University, Cheon-an 31116, Korea; minkoochoi@dankook.ac.kr; 2 BK21 FOUR Community-Based Intelligent Novel Drug Discovery Education Unit, Vessel-Organ Interaction Research Center (VOICE), Research Institute of Pharmaceutical Sciences, College of Pharmacy, Kyungpook National University, Daegu 41566, Korea

Due to the growing use of herbal supplementation—ease of taking herbal supplements with therapeutics drugs (i.e., does not require a prescription) and a trend of polypills with different modes of action for better therapeutic outcomes—there has been an increase in the rate of drug–drug interactions (DDIs), herb–drug interactions (HDIs), and adverse drug reactions. In the United States, approximately 76% of the adult population consumed herbal supplements in 2017; this increased by approximately 12% compared to 9 years ago, when it was still at 64%. Moreover, approximately 25% of herbal supplement users regularly take prescribed drugs, which increases the possibility of HDIs [1]. The most frequently reported HDIs include the modulation of herbal components on drug metabolizing enzymes and transporters as well as the causative pharmacokinetic alterations of co-administered therapeutic drugs as victim drugs. In this sense, the pharmacokinetic principles and DDI issues of victim drugs have been applied to the herbal supplements and their components. Therefore, it is important to highlight pharmacokinetic DDIs or HDIs and to understand their mechanisms in relation to the drug metabolizing enzymes and drug transporters.

The US Food and Drug Administration and European Medicines Agency guided in vitro and in vivo studies for the mechanistic understanding and quantitative prediction of clinical DDIs. In vitro DDI studies have assessed the inhibitory potential and inducibility of cytochrome P450 isozymes (CYPs) and uridine diphosphate-glucuronosyltransferases (UGTs) as well as drug transporters such as organic cation transporters (OCTs), organic anion transporters (OATs), organic anion transporting polypeptide (OATP), P-glycoprotein (P-gp), and breast cancer-resistant protein (BCRP).

The inhibition of CYP2D6 by berberine and fluoxetine has also been investigated [2,3]. These are the representative and clinically important drug-metabolizing enzymes with investigating in vitro inhibitory potential and a deeper mechanistic understanding. Berberine exhibited selective quasi-irreversible inhibition of CYP2D6 with an inactivation clearance of 5.83 mL/min/µmol. Thalifendine also exhibited time-dependent CYP2D6 inhibition. However, other berberine metabolites such as demethyleneberberine, demethylenethalifendine, and berberrubine showed non-selective and less potent inhibition of CYP2D6. This suggests that methylenedioxybenzene moiety may play a critical role in the quasi-irreversible inhibition. The coordinative potent and irreversible inhibition of berberine and its major metabolites may potentiate the possibility of berberine–drug interaction in the clinical setting [2].

S-fluoxetine and S-norfluoxetine are substrates for CYP2D6 with a strong CYP2D6 binding affinity; they reversibly inhibit CYP2D6 with inhibitory coefficient (K_i_) of 68 and 35 nM, respectively. Unbound steady-state plasma concentrations of S-fluoxetine and S-norfluoxetine higher than K_i_ values, with their long elimination half-life, are expected to contribute to prolonged DDIs on CYP2D6 activity. Moreover, fluoxetine is a substrate for CYP2C19 and exerts mechanism-based inhibition of this. Overall, fluoxetine and norfluoxetine are likely to be perpetrator drugs for CYP2C19 and CYP2D6 substrates. This is validated by an in vivo phase I clinical study, which showed reduced formation of the clopidogrel active metabolite (mediated largely by CYP2C19) and increased platelet aggregation when fluoxetine was coadministered with clopidogrel. It also possess the possibility of CYP2D6 substrates such as tramadol, codeine, β-blockers, class I antiarrhythmic drugs, first-generation H1-antagonists, and tricyclic antidepressants [3].

Once the potential single or multiple inhibitions of these metabolizing enzymes and transporters were identified, the quantitative prediction of DDIs between coadministered therapeutic drugs could be done using physiologically based pharmacokinetic modeling approaches. For this, information on the steady-state concentrations of perpetrator drugs or herbs after the administration of a high therapeutic dose, protein binding, and inhibitory coefficient is required. Because of difficulties regarding clinical pharmacokinetic data or in cases of herbal medicine with multiple components of similar structure and inhibitory potential, in vivo proof-of-concept study on experimental animals and phase I clinical study has been performed.

In relation to OAT6-mediated accumulation of sorafenib in keratinocytes and OATP1B1-mediated enterohepatic circulation of sorafenib-glucuronide, the influence of probenecid (an OAT and OATP inhibitor) on sorafenib pharmacokinetics and toxicity was investigated in 16 patients diagnosed with advanced hepatocellular carcinoma or differentiated thyroid carcinoma [4]. Patients received sorafenib (200–800 mg daily) in combination with probenecid (500 mg two times daily) for 2–15 days. Coadministered probenecid significantly decreased the concentrations of sorafenib in the plasma and keratinocytes but increased sorafenib-glucuronide concentrations; this may be because of the inhibitory effect of probenecid on OAT6 and OATP1B1 [4].

Lee et al. [5] evaluated the possible pharmacokinetic interactions between fenofibrate (peroxisome proliferator-activated receptor α agonist) and pitavastatin (3-hydoxy-3-methylglutaryl-coenzyme A reductase inhibitor) in healthy Korean subjects through an open-label, randomized, multiple-dose, three-period, and six-sequence crossover study with a 10-day washout in 24 healthy volunteers. To maximize the pharmacokinetic drug interaction potential, healthy subjects were separately and concomitantly administered high therapeutic doses of fenofibrate (160 mg) and pitavastatin (2 mg) once daily for 5 days. After pharmacokinetic comparison, there were no clinically significant pharmacokinetic interactions observed between micronized fenofibrate and pitavastatin when 160 mg of micronized fenofibrate and 2 mg of pitavastatin were co-administered. The treatments were well tolerated during the study, with no serious adverse events. Moreover, the frequency of increased creatinine phosphokinase, which was the most common adverse effect in this study, was similar whether the drugs were co-administered or administered separately as single agents.

Dutasteride, a 5α-reductase inhibitor, undergoes CYP3A4/3A5-mediated hepatic metabolism as a major elimination pathway. After comparison of metabolic activity, an inhibitory coefficient of ketoconazole on the metabolic activity of dutasteride and protein binding of dutasteride had the least species difference between rats and humans. During the coadministration of ketoconazole (20 mg/kg) with dutasteride, the area under plasma concentration curve (AUC) of dutasteride increased, whereas the AUC of its major active metabolite, 6β-hydroxydutasteride decreased following intravenous (2.5 mg/kg) and oral administration (5 mg/kg) of dutasteride in rats. These results are likely due to the decreased metabolic activity of dutasteride in the presence of ketoconazole [6].

Treatment with the active form of vitamin D3 (1α,25-dihydroxyvitamin D3) decreased the expression of Cyp2b and Cyp2C in rats and consequently decreased the intrinsic clearance of bupropion (which is hydroxylation-catalyzed by Cyp2b) and tolbutamide (which is hydroxylation-catalyzed by Cyp2c) in rat liver microsomes prepared from 1α,25-dihydroxyvitamin D3-treated rats. Since 1α,25-dihydroxyvitamin D3 did not inhibit the metabolic activity of bupropion and tobutamide in rat liver microsomes, the decreased metabolic intrinsic clearance of bupropion and tobutamide could be associated with the decreased Cyp2b and Cyp2C expressions. However, the in vitro effect of 1α,25-dihydroxyvitamin D3 on Cyp2b and Cyp2C does not directly translate into the pharmacokinetics of bupropion and tobutamide and their metabolites hydroxybupropion and hydroxytobutamide because of the complicated regulation of 1α,25-dihydroxyvitamin D3 on renal function, protein binding, and other transport activities [7]. A lack of in vitro and in vivo correlation was also reported by Neag et al. [8]. Grape pomace extract did not inhibit the renal uptake of cisplatin mediated by cation transporters, which play important roles in cisplatin toxicity. However, grape pomace extract significantly increased blood creatinine and urea levels, the severity of kidney histopathological damage, and mortality in all cisplatin groups. Therefore, the underlying mechanism of nephrotoxicity enhancement by grape pomace extract warrants further investigation based on individual components and their actions. On the other hand, repeated administration of mulberry leaf extract for 3 weeks enhanced the hypoglycemic efficacy of metformin by 49% compared to metformin monotherapy in streptozotocin-induced diabetic rats. The potentiated hypoglycemic effect could be attributed to the increased metformin exposure by the intake of mulberry leaf extract, which is caused by the decreased renal clearance of metformin mediated by the OCT2 transporter in the presence of mulberry leaf extract [9]. However, the role of trans-caffeic acid, major component in mulberry leaves extract (0.7 mg/g), as well as other components in the HDI warrants further investigation.

Tran et al. [10] investigated the effect of galgeuntang on the pharmacokinetics of acetaminophen in 12 healthy male subjects using a population pharmacokinetics modeling approach. The pharmacokinetics of acetaminophen is best described as one-compartment with first-order elimination and two-period absorption phases. Overall, coadministration of galgeuntang slightly decreased the AUC of acetaminophen without changing the apparent clearance. The intake of the highest dose of galgeuntang decreased the absorption rate constant of acetaminophen by increasing the mean residence time of acetaminophen from the stomach to the small intestine, which resulted in the decrease in maximum plasma concentration (C_max_) and AUC of acetaminophen compared with the administration of acetaminophen alone. Overall, coadministration of galgeuntang slightly decreased the C_max_ and AUC of acetaminophen without changing its apparent clearance.

In addition to the possibility of HDIs between herbal supplements and therapeutic drugs, the mechanistic understanding regarding the individual component is also important. This special issue focused on the multifaceted factors causing conflicting outcomes in HDIs [11]. In summary, the chemical constituents in herbal supplements vary according to cultivation area, harvest time, storage condition, and preparation methods (i.e., use of ethanol extraction, etc.) [12]. This emphasizes the importance of standardized materials along with the quantitative analysis of main and effective components [1,13]. Moreover, several bioactive constituents in one herbal supplement may interact with therapeutic drugs due to their similar inhibitory characteristics, but the pharmacokinetic features of these bioactive components have not yet been fully investigated [14]. The treatment period, route of administration, and dose of herbal supplements are also important. The unabsorbed constituents in orally administered herbal extracts could not be involved in in vivo interactions in the hepatic metabolism of a coadministered drug. However, all constituents in a herbal extract are tested in in vitro hepatocytes and liver microsomes, even though some could not reach the liver due to the lack of absorption in vivo [11]. Under certain circumstances, such as when multiple components simultaneously inhibit the drug-metabolizing enzymes and transporters but the pharmacokinetics of these components are not yet fully characterized, an in vivo cocktail approach may have advantages to efficiently evaluate potential DDIs or HDIs. Kwon et al. [15] designed a two-step validation process to develop and validate the dual cocktail including composed of caffeine (1 mg/kg), diclofenac (2 mg/kg), omeprazole (2 mg/kg), dextromethorphan (10 mg/kg), nifedipine (0.5 mg/kg), metformin (0.5 mg/kg), furosemide (0.1 mg/kg), valsartan (0.2 mg/kg), digoxin (2 mg/kg), and methotrexate (0.5 mg/kg).

Clinical data regarding DDIs and HDIs from medical databases analyzed retrospectively can reveal important information especially for patients with cardiovascular disease who take many different medications. Spanakis et al. [16] analyzed the DDIs and HDIs in 76 patients undergoing cardiothoracic surgery. Among the 166 DDIs, 32% were related to pharmacokinetics (PK-DDIs), whereas 68% were related to pharmacodynamics (PD-DDIs). The frequency of PK-DDIs was higher during admission and discharge, whereas PD-DDIs were mainly recorded during preoperation and postoperation periods [17,18]. Austero et al. [17] performed a systematic review regarding the potential HDIs during the management of age-related cognitive dysfunction. They included 170 bioactive herbal components used by seniors for cognitive enhancement and 10 pharmaceuticals commonly prescribed to middle-aged adults. Cognitive-enhancing functional phytochemicals included alkaloids (25%), terpenoids (21%), flavonoids (20%), phenolic acids (12%), and others. The most affected targets by these phytochemicals are CYP3A4 (39%), P-gp (32%), COX2 (28%), CYP2C9 (28%), CYP1A2 (26%), and BCRP (18%) among others.

Overall, all the papers in this special issue emphasized the significance of pharmacokinetic DDIs and HDIs and their possible mechanisms along with commonly used therapeutic drugs and herbal supplements. Moreover, the mechanisms of DDIs and HDIs could share the same targets even though herbal supplements and therapeutic drugs have diverse chemical structures. The predicted DDIs and HDIs with therapeutic drugs should be considered with care by healthcare professionals for alternative therapeutic regimens to be provided.

## Data Availability

Not applicable.
